# Effect of Arsenic Stress on Expression Pattern of a Rice Specific *miR156j* at Various Developmental Stages and Their Allied Co-expression Target Networks

**DOI:** 10.3389/fpls.2020.00752

**Published:** 2020-06-16

**Authors:** Akhilesh Kumar Pandey, Mallikarjuna Rao Gedda, Ashok K. Verma

**Affiliations:** ^1^Department of Biochemistry, Institute of Science, Banaras Hindu University, Varanasi, India; ^2^Biotechnology Laboratory, U.P. Council of Sugarcane Research, Shahjahanpur, India

**Keywords:** rice, arsenic, microRNA, *Osa-miR156j*, gene expression, developmental stage

## Abstract

In plants, arsenic (As) stress modulates metabolic cascades at various developmental stages by influencing the pattern of gene expressions mediated by small non-coding RNAs, especially Micro-RNAs, involved in the moderation of a myriad of cellular processes needed for plant adaptation upon oxidative stress. *miR156j* of *miR156* gene family, involved mainly in the regulation of growth and development in plants. This study was designed to find out the role of arsenic toxicity on *Osa-miR156j* expression in all physiological growth stages. To better understand the functional role of *Osa-miR156j* in rice, we observed the expression in different developmental stages (seedlings, tillering and flowering) and various tissues of leaf, stem and root tissues (at 0, 24, 48, and 72 h) under 25 μM arsenite [As (III)] exposure. Additionally, using bioinformatic tools to target genes of *Osa-miR156j* and the potential co-expressed genes were explored at different development stages in the various tissues of rice under stress conditions. The expression of *Osa-miR156j* showed its temporal downregulation in various tissues in different developmental stages. Of note, the downregulation was more pronounced in root tissues at seedlings, tillering, and flowering stages during 0–72 h under arsenite exposure as compared to other tissues. Overall, the As stress altered the gene expression more prominently at seedlings developmental stage followed by flowering and tillering. Additionally, through the *In silico* approach, the target functions and presence of oxidative stress-responsive cis-acting regulatory elements/motifs also confirmed *Osa-miR156j* involvement in the regulation of arsenic stress in rice. The findings of this study demonstrate the prominent role of *Osa-miR156j* in rice under arsenite stress, which was found to modulate the metabolic activities in rice plants at different developmental stages, and thus it might be useful for the development of arsenic tolerant varieties.

## Introduction

Arsenic (As) is a metalloid, which is ubiquitously distributed in soils, most plants species, and aquifers (Zhang and Selim, 2006). This metalloid can cause some severe threats to human health primarily through biomagnification in the food chain due to contaminated crops (Meharg and Hartley-Whitaker, 2002). It’s been estimated that an average natural arsenic content level is about 5 mg/kg in the soils worldwide, which is due to leaching from sedimentary rocks, mining and smelting of the As bearing ores and minerals as well as the coal mining (Le Hécho and Matera, 2001; Francesconi et al., 2002; Martin et al., 2014). However, due to the indiscriminate use of arsenic in herbicides, insecticides, and wood preservatives, etc., its concentration is increasing at an alarming rate in the soil of South-East Asian sub-continent (Shankar and Shanker, 2014). Rice (*Oryza sativa*) is the staple food and hence, principal crop for over half of the world. The presence of high arsenic content in the soil has led to its accumulation in paddy, which has resulted in its collection at the concentration beyond the food safety threshold (Zhu et al., 2008; Zheng et al., 2011). Arsenic accumulation in plants culminates in both direct and indirect toxicity (Verbruggen et al., 2009). Some studies on arsenic toxicity on rice seedlings showed compromised photosynthetic rate (Stoeva et al., 2003; Gautam et al., 2019), disturbed carbohydrate metabolism (Jha and Dubey, 2005), subdued nitrogen assimilation (Jha and Dubey, 2004), elevated phytochelatin synthesis (Mishra et al., 2011), overproduction of reactive oxygen species (ROS), marked increase in lipid peroxidation in it leading to the oxidative stress (Choudhury et al., 2011; Mishra et al., 2011; Gautam et al., 2020). However, plants have an array of mechanisms to detoxify arsenic poisoning, which mainly include metal transport, chelation, and sequestration through organoarsenic compounds (Verbruggen et al., 2009; Mishra et al., 2011; Chen et al., 2017; Pandey et al., 2019).

MicroRNAs (*miRNAs*) are a discrete class of short-chain (21–24 nucleotides) small RNAs that play a central role in modulating gene expression at post-transcriptional levels (Bartel, 2004). The *miRNAs* have been reported to play an essential role in plant tolerance to abiotic stresses such as drought, salinity, cold, heavy metal toxicity, and nutrient deprivation (Moldovan et al., 2009). A variety of *miRNAs* have been identified and characterized for various metabolic and biological processes, including tissue maturation (Mlotshwa et al., 2006), signal transduction (Vernoux and Benfey, 2005), hormone signaling (Eckardt, 2005), the phase change from vegetative to reproductive growth (Lauter et al., 2005), and response to environmental stresses (Sunkar and Zhu, 2004). The plant *miR156* has been reported to target squamosa promoter binding protein-like (SPL) transcription factor family (Gou et al., 2011), which are expressed more at the younger phase whose expression gradually declines with the plant age (Matts et al., 2010). In *Arabidopsis*, *miR156* plays a critical role during different developmental stages such as leaf development, shoot maturation, phase change, and flowering by targeting 11 SPLs out of 17 SPL genes. The over-expression of *miR156* in transgenic *Arabidopsis* showed reduced apical dominance, delayed flowering time, caused dwarfism, and increased total leaf numbers and biomass (Schwab et al., 2005). Out of 19 SPLs, *Osa-miR156* targets 11 SPLs, which influence various morphological changes during the development of rice (Xie et al., 2006). Some reports suggest its crucial roles during various abiotic stresses (Khraiwesh et al., 2012; Sunkar et al., 2012). A recent report from Yu et al. (2012) pointed out the inevitable role of *Osa-miR156j*, a member of the *Osa-miR156* family, due to its significant down-regulation in rice seedlings during arsenic-stress. This down-regulation suggests some important regulatory functions associated with *Osa-miR156j* during arsenic stress at developmental stages in rice. Therefore, this study was planned to extricate the effect of arsenic stress on the expression of *Osa-miR156j* at different developmental stages in different tissues for better understanding of *miRNA* functional genomics and the molecular role of involved genes against As-induced stress tolerance in rice.

## Materials and Methods

### Rice Growth and Arsenic Treatment

Rice (*Oryza sativa* L.) cultivar IR-64 seeds were germinated in a soil-less medium. Seeds were surface-sterilized with 3% sodium hypochlorite solution for 20 min and washed with distilled water. The sterilized seeds were soaked in distilled water for 24 h in a flask at 37°C. Soaked seeds were kept on moistened filter paper in Petri dish and incubated at 30°C under the dark condition for three days for germination. After germination, the coleoptile of germinated seeds was slotted in the styrofoam and placed in Yoshida nutrient solution (YS) (Yoshida et al., 1971) containing disposable 250 ml plastic cups. For the natural growth of the seedlings, the hydroponic system was transferred in glasshouse conditions with day/night temperatures of 30/20°C and relative humidity of at least 50% during the day. The 14 days old seedlings were treated with 25 μM arsenite (NaAsO_2_). For the treatments of arsenite in tillering and flowering stages, 14 days old fresh seedlings were subsequently transferred to soil-filled pots containing balanced nutrients as per requirement. After attaining the tillering stage (27 days) and flowering stage (60 days), plants were harvested with roots. The roots were washed with tap water. For the treatment of arsenite, plants were subjected to hydroponic media containing 25 μM arsenite. At all the developmental stages, plant samples were collected at 0, 24, 48, and 72 h from leaf, stem, and roots. The samples were frozen immediately in liquid nitrogen and stored at -80°C until used for further analysis.

### Targets and *cis*-Elements/Motif Prediction

To find out targets of *Osa-miR156j*, the publically available tool psRNATarget^1^ was used by searching cDNA OSA1 release five and transcript, RAP-DB, version 1.0 as reference genome (Dai and Zhao, 2011). To identify the molecular functions of the searched targets, we used the AgriGO^2^ online analysis tool (Du et al., 2010). The upstream sequence 200-bp was analyzed by PlantCARE^3^ to search for the presence of *cis*-regulatory elements/motifs (Lescot et al., 2002).

### *In silico* Co-expression Network and MPSS Analysis of Target Genes

The Gene co-expression network of *miRNA*-target genes and co-expressed genes were obtained from the publically available tool RiceFrend^4^, and networks were constructed using Cytoscape 3.2.1 tool (Praneenararat et al., 2012; Tiwari et al., 2017). Massively Parallel Signature Sequencing (MPSS) data were obtained from the rice MPSS database for different development stages and various tissues of Rice. Collected data were further analyzed by the Clustvis tool^5^.

### RNA Extraction, Polyadenylation, and cDNA Synthesis

Total RNA in collected samples was extracted by using TRIzol^®^ reagent (Invitrogen) according to the manufacturer’s instructions. The quality of extracted RNA was visually examined by the 28S/18S ribosomal bands on an agarose gel, and the quantity was checked by spectrophotometer. Total RNA and Short length of *miRNA* were treated with *E. coli* poly A polymerase (NEB, United Kingdom) to generate a poly-A tail at the 3′ end of each RNA molecule. The polyadenylation reaction with a final volume of 20 μl includes 250 ng of total RNA, 4.0 μl of 5 × poly-A polymerase buffer, 1.0 μl of 10 mM rATP and 1 μl of *E. coli* poly A polymerase (New England Biolabs, United States). The reaction was incubated at 37°C for 30 min and adenylation terminated by incubating reaction at 95°C for 5 min. The1st-strand cDNA synthesis was performed using a *miRNA* 1st-strand cDNA synthesis kit (Stratagene) as per manufacturer’s instructions.

### Primers Design and qRT-PCR Analysis

The DNA primers used in real-time analysis of *Osa-miR156j* were designed as previously described by Balcells et al. (2011). Reference gene, β-Actin (Wu et al., 2009; Peng et al., 2013) primers were designed using Primer Express^®^Software v2.0 (Applied Biosystems, United States) under default parameters. The primer sequences used in this study are given in Supplementary Table S1. For quantification of *miRNA*, final reaction, 25 μl was comprised 2 μl of cDNA (10 ng), 12.5 μl of 2× SYBR advantage premix, 0.5 μl of 50× ROX dye (Clontech, United States), 0.5 μl of 10 μM *miRNA* specific primers. A negative control without a template, the reaction was also performed with the same primers. Three biological replicates for each sample were used for RT-PCR analysis. The real-time PCR was performed, employing a 7300 Real-Time PCR System (Applied Biosystems, United States). PCR cycles 95°C for 10 s, followed by 35 cycles of 95°C for 10 s and 60°C for 20 s, were performed in 96-well optical reaction plates (Applied Biosystems). The specificity of the amplicon was observed by analysis of the melting curve after 35 cycles at 60–90°C. The abundance/decline of *Osa-miR156j* was normalized to a geometric average of endogenous control (β-actin) for ΔCt and expressed as relative *Osa-miR156j* expression.

### Northern Blotting Analysis

Total RNA from different samples was isolated with Trizol reagent. The total RNA, 25 μg, was resolved in the per lane on a denaturing 15% polyacrylamide gel with 7% urea at 40 mA for 2 h. The gel was stained with ethidium bromide, and 5S RNA/tRNA bands were used as loading controls. RNAs were electro-blotted on Hybond N + membranes (Amersham Biosciences, NJ) using a Trans-Blot Cell (Bio-Rad, CA, United States). Membranes were UV cross-linked and baked at 80°C for 1 h. DNA oligonucleotide probe complementary to *miRNA* was labeled with ATP γ-P^32^ using T4 polynucleotide kinase (NEB, United Kingdom). The blot was pre-hybridized for 30 min at 40°C in pre-hybridization buffer containing 7% SDS, 200 mM Na_2_HPO_4_ (pH 7.0), 5 μg/ml salmon sperm DNA. After removing the pre-hybridization buffer, the blot was hybridized with a 50 pmol/ml probe containing a hybridization buffer for 16 h at 40°C. The hybridized blot was subsequently washed three times with 1X SSC, 0.1% SDS buffer at room temperature. Finally, the blot was exposed to a phosphor screen and cross-linked with 1200 μ joules for 20 min to improve sensitivity. The bands were analyzed using the Alpha Imager documentation system. The data were expressed as mean ± standard deviation (SD) of the band density of the experiments.

### Statistical Analysis

All the experiments were performed in triplicate and repeated three times, and the statistical analysis between the different groups was performed by Student *t*-test using GraphPad Prism 7.0 software. The data were represented as mean ± SD with *p-*value < 0.05 was considered significant.

## Results

### Identification of Potential Targets and *cis*-Regulatory Elements/Motifs in *Osa-miR156j* and Their Co-expression Gene Networks and MPSS

**FIGURE 1:**
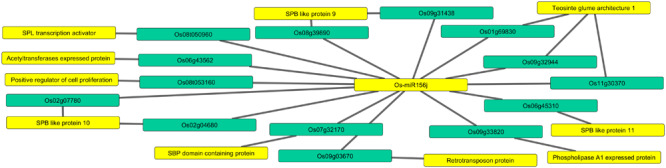
Predicting network of *Osa-miR156j* potential target genes (locus IDs) and their target functions. The miRNA and essential target functions linked to arsenic stress such as squamosa promoter binding like protein, teosinte glume architecture 1, Phospholipase A1 activity, retrotransposon protein, etc., are highlighted in yellow color, and the targeted genes in sea green color.

**FIGURE 2:**
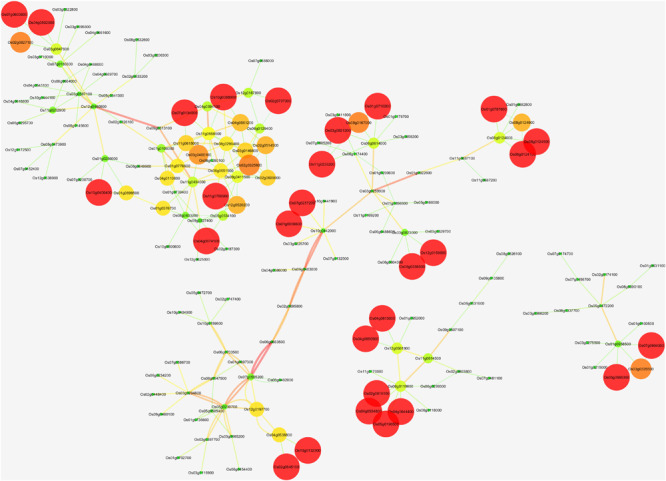
The network of *Osa-miR156j* target gene; Os01g0922600, Os06g0663500, Os07g0505200, Os09g0507100, Os02g0174100, and Os09g0513100, their potential co-expressed target genes using Cytoscape 3.2.1 software. Visualization parameters were based on the clustering coefficient with low values to the small size of the nodes.

The potential targets of *Osa-miR156j* (locus IDs), mainly comprised of DNA binding transcription factors and DNA binding proteins, acetyltransferases, phospholipase A1, teosinte glume architecture 1, etc., as shown in Figure 1 and Supplementary Table S2. Furthermore, *Osa-miR156j* co-expression target networks are shown in Figure 2 and Supplementary Table S3. To elucidate *cis*-acting regulatory elements linked with the regulation of diverse metabolic and physiological functions, the immature sequence of *Osa-miR156j* was analyzed using the bioinformatics tool, PlantCARE. The potential *cis*-acting regulatory elements in the promoter region of *miRNA* were comprised with the presence of many common abiotic stress regulatory motifs/elements like ABRE (abscisic acid responsiveness), CCAAT-box (MYB binding site), GC-motif (enhancer like elements involved in anoxic specific inducibility), HSE (heat stress-responsive elements), MSA (involved in cell cycle regulation response) and TCA (salicylic acid responsiveness).

**FIGURE 3:**
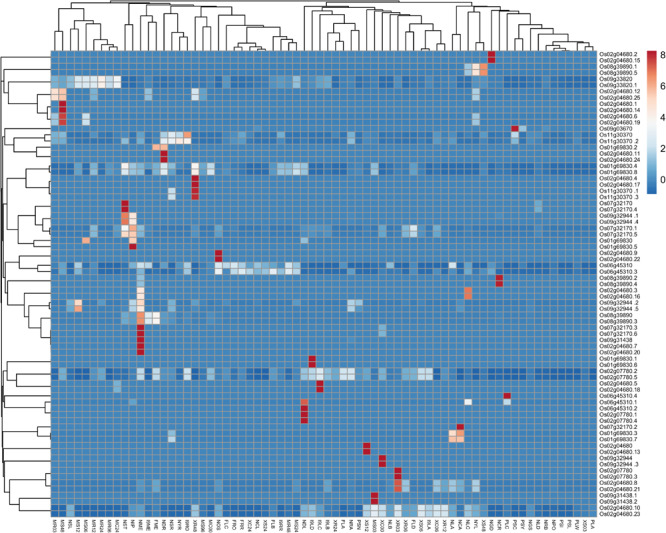
Heat map generated from MPSS data using the Clustvis tool shows the *in silico* expression level and classification of identified genes at different development stages, various tissues under biotic, and abiotic stress in rice. Expression values obtained from Rice MPSS for 17 and 20 bp sequences were used for Heat Map generation. NYR; 14 days young roots, NRA; 60 days mature roots replicate A, NRB; 60 days mature roots replicate B, NGD; 10 days germinating seedlings in the dark, NST; 60 days stem, NYL; 14 days young leaves, NLA; 60 days mature leaves replicate A, NLB; 60 days mature leaves replicate B, NLC; 60 days mature leaves replicate C, NLD; 60 days mature leaves replicate D, NME; 60 days crown vegetative meristematic tissue; NPO; Mature pollen, NOS; ovary and mature stigma, NIP; 90 days immature panicle, NGS; 3 days germinating seed, NCA; 35 day callus, NSR; 14 days young roots stressed in 250 mM NaCl for 24 h, NSL; 14 days young leaves stressed in 250 mM NaCl for 24 h, NDR; 14 days young roots in drought for 5 days, NDL; 14 days young leaves in drought for 5 days, NCR; 14 days young roots stressed at 4°C for 24 h, NCL; 14 days young leaves stressed at 4°C for 24 h, XC00; unwounded Nipponbare Xa-21 0 h, XC06; mock treatment 6 h, XC24; mock treatment 24 h, XR03; X. oryza R 3 h, XR06; X. oryza R 6 h, XR12; X. oryza R 12 h, XR24; X. oryza R 24 h, XR48; X. oryza R 48 h, XS03; X. oryza S 3 h, XS06; X. oryza S 6 h, XS12; X. oryza S 12 h, XS24; X. oryza S 24 h, XS48; X. oryza S 48 h, MR03; M. grisea R 3 h, MR06; M. grisea R 6 h, MR12; M. grisea R 12 h, MR24; M. grisea R 24 h, MR48; M. grisea R 48 h, MS03; M. grisea S 3 h, MS06; M. grisea S 6 h, MS12; M. grisea S 12 h, MS24; M. grisea S 24 h, MS48; M. grisea S 48 h, MS96; M. grisea S 96 h, MC00; mock treatment 0 h,MC24; mock treatment 24 h,I9RO; roots, I9RR; roots replicate, I9LA; leaves, I9LB; leaves replicate,I9LC; leaves, I9LD; leaves replicate, I9ME; Meristematic tissue, FRO; F1 hybrid 60 days mature roots, FRR; F1 hybrid 60 days mature roots replicate, FLA; FLA; F1 hybrid 60 days mature leaves replicate A, FLB; F1 hybrid 60 days mature leaves replicate B, FLC; F1 hybrid 60 days mature leaves replicate C,FLD; F1 hybrid 60 days mature leaves replicate D, FME; F1 hybrid 60 days meristematic tissue, PSC; rice developing seeds 6 days old cypress high milling (99-1710), PSI; rice developing seeds Il pumbyeo high taste, PSL; rice developing seeds 6 days old Lagrue low milling, PSN; rice developing seeds 6 days old Nipponbare grain quality control, PSY; rice developing seeds 6 days old YR15965Acp33 low taste, PLA; rice leaf armyworm damaged 24 h (99-1726), PLW; rice leaf water weevil damaged 24 h, PLC; rice leaf mechanically damaged 24 h.

Using MPSS data, Heat map generated using the Clustvis tool shows *in silico* expression level and classification of identified genes at different development stages, various tissues under biotic and abiotic stress in rice (Figure 3 and Supplementary Table S4). Further, the expression values obtained from Rice MPSS for 17 and 20 bp sequences were used for analysis and representation. Red color designates the highest level of expression frequency at different development stages and various tissues followed by other colors for their corresponding level of expression in the heat map.

### Expression Analysis by RT-PCR and Northern Blotting in Various Plant Tissues

**FIGURE 4:**
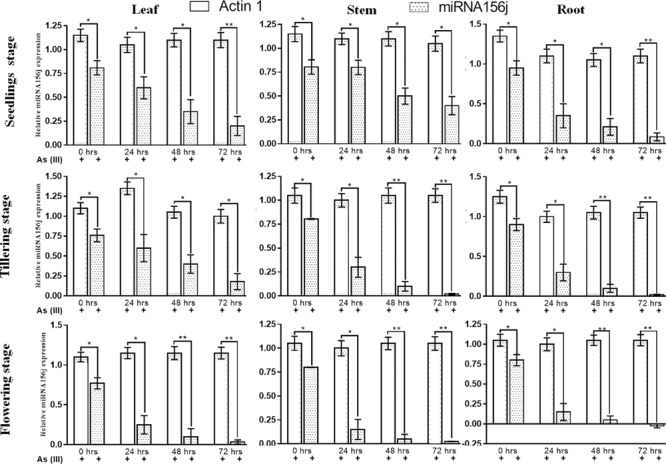
Real-time PCR expression analysis of arsenic-induced *miR156j* expression at different time intervals (0–72 h) and various developmental stages (seedlings, tillering, and flowering) in rice leaf, stem, and root tissues. The qPCR experiments were done in triplicate, and the relative *miR156j* expression data at different developmental stages and different tissues in rice were represented as mean ± SD with *p < 0.05, **p < 0.01, ***p < 0.001 as significant.

To investigate the role of *Osa-miR156j* under As stress at various developmental stages, the expression patterns were studied in different tissues of seedlings, tillering, and flowering physiological stages in rice during 0–72 h. The results show that the expression of *Osa-miR156j* at the seedling stage in the stem was a nearly similar or slight decline in expression at 48 and 72 h exposure (Figure 4). Up to 24 h of As (III) treatment, there was no change in expression, while 1.61 and two-fold decline in expression of *Osa-miR156j* was observed in 48 and 72 h exposure. In the leaf of the seedling stage, a 1.8-fold decline in expression at 24 h, followed by a 2.31 and 4.05-fold decline in expression of *Osa-miR156j* at 48 and 72 h exposure was recorded. The expression of *Osa-miR156j* at the seedling stage in root followed the same pattern as the leaf. The 2.71, 4.52, and 11.17-fold decline in expression was observed at 24, 48, and 72 h As (III) exposure, respectively. At the tillering stage, the expression of the *Osa-miR156j* in leaf declined only by 1.26-fold, followed by 1.9-fold at 24 and 48 h exposed plants. The 4.22-fold decrease in *Osa-miR156j* expression was recorded after 72 h As (III) exposure. But at the tillering phase, a sharp decline in expression *Osa-miR156j* was observed, i.e., 8.05, followed by 40.25-fold in 48 and 72 h exposed plant stems, respectively. In the root of tillering phase 3, 9, and 45-fold decline in expression of *Osa-miR156j* was recorded at 24, 48, and 72 h exposed to As (III), respectively. While at flowering stages, the sharp decline in expression of the *Osa-miR156j* gene was recorded with an increase in As (III) exposure time. The expression of *Osa-miR156j* at the flowering stage in leaf tissue is lower in comparison to the stem and root cells of plants. The expression of the *Osa-miR156j* in leaf declined by 3.08, followed by 7.70 and 22-fold at 24, 48, and 72 h As (III) exposure, respectively. Similar results were obtained in stem and root cells as nearly 5.5-fold decline in expression of *Osa-miR156j* exposed to 24 h, while 16-fold decline in stem and root cells exposed to 48 h As (III) exposure.

**FIGURE 5:**
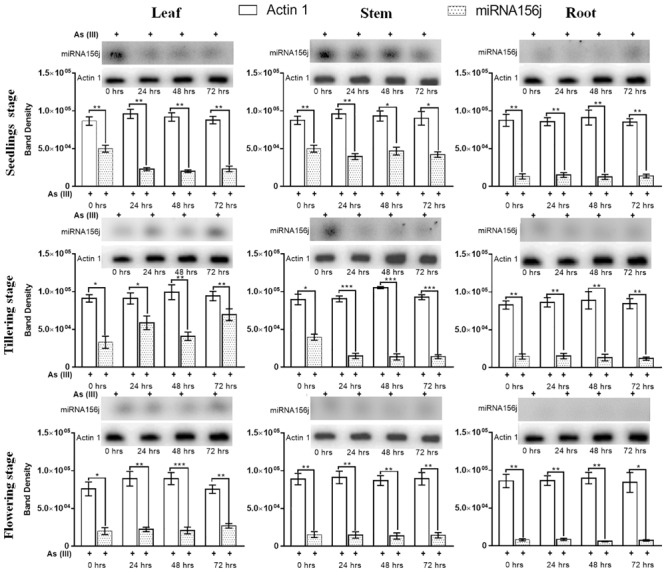
Arsenic induced *Osa-miR156j* expression by Northern blotting at different developmental stages (seedlings, tillering, and flowering) in rice leaf, stem, and root. The Northern blotting experiments were done in triplicate, and the band density data at different developmental stages and different tissues in rice were represented as mean ± SD with *p < 0.05, **p < 0.01, ***p < 0.001 as significant.

For further validation of the expression results obtained, we used Northern blotting analysis to examine expression levels. Northern blotting analysis gave a similar analysis pattern in agreement with the RT-PCR results (Figure 5). At 0th h in the leaf, stem, and root at all developmental stages under arsenic stress, we observed *Osa-miR156j* significant (*p* < 0.05) decreased band density. This has also been seen in 24, 48, and 72 h, with a constant significant decrease in the band density levels of *Osa-miR156j* under As (III) with 72 h showing the least band density in the seedlings, tillering, and flowering stages. The duration of As (III) exposure was also found to affect the gene expression such that with an increase in exposure time, the downregulation of the expression of *Osa-miR156j* was more pronounced.

## Discussion

*Oryza sativa* is the primary cereal crop, and its productivity is compromised under several abiotic stresses such as heavy metals, heat, salt, and drought, etc. The distribution of metalloid arsenic is ubiquitous in the environment, which is reported to cause severe stress in rice. Studies by Chowdhury et al., 2018 and Das et al., 2013 have shown the accumulation of As at maximum levels in roots as compared to leaves and stems, as the dosage increases As accumulation increases in a similar manner (Das et al., 2013; Chowdhury et al., 2018). These accumulations occur maximum in the roots followed by stem than in the leaves and least in the economic production. Besides, it is said to get accumulated in the edible parts, i.e., grains (2 mg/kg) and present a noteworthy illustration of biomagnification by finally reaching the human food chain where they exhibit several deleterious effects (Williams et al., 2005; Li et al., 2016). Arsenic in the form of arsenite, i.e., As (III) enters plant by the roots through nodulin 26-like intrinsic protein (aquaporins) (Li et al., 2014; Mukhopadhyay et al., 2014). Studies in rice plants have shown that aquaporins (OsNIP1;1, OsNIP3;1, OsNIP3;2, OsNIP3;3, OsNIP2;1, OsNIP2;2, OsLsi2, OsPIP2;4, OsPIP2;6 etc.) help in the uptake, transport, and influx of As (III) (Bienert et al., 2008; Ma et al., 2008; Katsuhara et al., 2014; Li et al., 2016; Khan and Gupta, 2018).

Albeit our substantial progress in understanding heavy metal(s) responses in plants, the molecular parameters responsible for physiological responses are still not well identified. Under metal toxicity, plants try to modify gene expression through *miRNAs* to regulate excess metals accumulation by various means such as complexation, chelation, antioxidant defense against ROS, and control multiple biological responses through signal transduction (Gielen et al., 2012). In response to As stress, plants modulate metabolic cascades at various developmental stages through alteration in gene expression. Therefore, it becomes prudent to understand the As-induced abiotic stress at the molecular level so that we may develop a strategy to curb this menace, or we can develop As-resistant rice variety. Plant *miRNAs* are involved in various stress responses that regulate the gene expression by negatively regulating the complementary target genes.

There are different microRNAs reported in plants with various functions, in which *miR156* has significant agricultural importance for biotic stress tolerance and plant development (Jones-Rhoades and Bartel, 2004). The genomic investigation of *miR156* deciphered that *miR156* regulatory repertoire is highly conserved during plant evolution (Axtell et al., 2007; Huijser and Schmid, 2011; Zhang et al., 2015). Schwarz et al., 2008; Xing et al., 2010 have reported that *miR156* downregulates *Squamosa Promoter Binding Protein Like* genes, which enrich growth from juvenile to mature, leaf formation, flowering, and vegetative phase (Schwarz et al., 2008; Xing et al., 2010). Sun et al., 2015 reported that all the conserved *miRNAs*, such as *miR156*, *miR171*, *miR172*, *miR395*, *miR397*, and *miR398*, were down-regulated in grapevine under abiotic stress (Sun et al., 2015). Yu et al., 2012 also reported that *miR156*, among other 12 *miRNA* families, plays a key role during the As stress in rice (Yu et al., 2012). Tang et al., 2019 also deciphered the non-coding RNA-level response to arsenic stress in rice (*Oryza sativa*) and enlarge the present molecular understanding of As stress response, particularly at the non-coding RNA level (Tang et al., 2019). The *miR156j*, a member of the *miR156* gene family, is highly conserved and helps in regulating the growth and development in plants. This study emphases the response of *Osa-miR156j* in rice cultivars exposed to As stress. Rice is an extensively studied model system in plant biotechnology; yet, the response of these *miRNAs* against As stress at the different growth phases of rice plant (seedling, tillering and flowering) on root leaf and stem separately has not been studied until our report. The scope of this study could be utilized for a better understanding of molecular mechanisms of arsenic stress and the roles of *miRNAs*. In the present study, we have investigated the expression pattern of *Osa-miR156j* and their associated target networks in different developmental stages in various tissues of rice during As stress.

*Osa-miR156j* is one of the more predominant plant microRNA families predicted to target SPL transcription factors to control developmental timing, and the phase change from vegetative to reproductive growth (Wu and Poethig, 2006). The candidates of this family are expressed mainly in the juvenile stage and gradually decrease with the advancement of the plant age (Matts et al., 2010). Identification of micro RNAs and their targets in switchgrass, a model biofuel plant species, target analysis of *Osa-miR156j* showed that its cleavage targets are *SPL9*, *SPL10*, and *SPL12* genes. During the growth phase, *SPL9* acts as a transcriptional repressor (Gou et al., 2011). Genes *SPL10* and *SPL11* play a central role in the development of lateral organs in association with shoot maturation at the reproduction stage, but SPL10 is the only transcription factor that controls shoot development regulation during vegetative growth (Shikata et al., 2009). Although, the expression of *Osa-miR156j* has been studied previously at seedlings stage in some plants including rice under arsenic stress (Srivastava et al., 2012; Yu et al., 2012) however, its expression profile (and hence the role) is entirely unknown at other developmental stages in different plant tissues at different time intervals. Taken together, our data suggest that the regulation of *Osa-miR156j* expression is highly influenced by SPLs transcription factors, acetyltransferases/phospholipase A1 enzymes, and its down-regulation might be results of activation/deactivation of these transcription factors/enzymes. In previous studies, these target genes have been reported for their involvement in plant growth and development during oxidative stresses (Wang et al., 2009; Khraiwesh et al., 2012). Acetyltransferases play a vital role in histone modification by acetylation during regulation of gene expression by DNA replication, DNA repair, DNA recombination, and gene transcription (Balcells et al., 2011; Liu et al., 2012). Histone acetylation by acetyltransferases (HATs) activates gene expression, whereas de-acetylation leads to gene repression (Chen and Tian, 2007, Gautam et al., 2020). In rice, HATs (OsHATs) are expressed constitutively, and their expression regulated by hormones and oxidative stresses (Liu et al., 2012). In *Arabidopsis*, HATs (AtHATs) have been reported to regulate light-inducible gene expression (Servet et al., 2010), root stem-cell niche maintenance (Kornet and Scheres, 2009) and regulation of *miRNAs* accumulation at transcriptional and posttranscriptional levels (Kim et al., 2009). Phospholipases are phospholipid hydrolyzing enzymes, which activate plant immune responses associated with the development of hypersensitive cell death (Jones and Dangl, 2006). These enzymes are known for the production of outstanding defense signaling molecules like oxylipins, jasmonates, and phosphatidic acid in plants (Canonne et al., 2011). In plants, members of the phospholipases super-family, phospholipase A1 plays an essential role in maintenance and remodeling cell membrane and regulates various cellular mechanisms by the production of various lysophospholipid mediators, such as lysophosphatidylserine and lysophosphatidic acid, which involve in multiple biological functions (Richmond and Smith, 2011).

In recent years, independent research on receptor-like protein kinases (RLKs) has shown its role in biotic and abiotic stress response and its resistance besides signal transduction in plants, which can further promote agriculture development (Ye et al., 2017). Our results through *In silico* networking have shown that *Osa-miR156j* is associated with eight potential genes. These identified potential genes (Os08g0509600, Os02g0174100, Os02g0139400, Os06g0663500, Os09g0513100, Os09g0507100,and Os01g0922600) were associated with zinc finger proteins, wall-associated receptor kinase, lectin receptor-like kinase, UDP-glycosyltransferase and lipid metabolic process, etc. through co-expression networks, which were analogous to the previous studies (Zhou et al., 2011; Yu et al., 2012; Cheng et al., 2013; Moon et al., 2013; Fu et al., 2014; Bellande et al., 2017). The presence of *cis*-regulatory elements in the promoter region indicated that *Osa-miR156j* might have an essential role in the regulation of transcription factors, metabolisms, stress responses, signaling transductions, cellular structural components, and other cellular processes. ABRE *cis*-regulatory elements play a vital role in regulating oxidative stresses in rice (Yamaguchi-Shinozaki and Shinozaki, 2006). Promoter elements ABREs are known to be controlled by phytohormone abscisic acid (ABA) and salicylic acid signaling (Gautam et al., 2020). They contribute to a range of developmental and adaptive processes to environmental stimuli in plants (Fujita et al., 2011). With an increase in endogenous ABA levels in response to osmotic stresses, ABA activates the expression of many genes through ABREs in their promoter regions (Fujita et al., 2013). HSEs induce oxidative stress genes and play an essential role during signal transduction pathways mediating activation of high temperatures and other abiotic stresses cold and heavy metals (Yi and Liu, 2009; Petrov et al., 2015). MSAs are M specific activators, and they control the M- phase-specific gene expression in plants (Ito et al., 1998). Hence, the presence of these *cis*-acting elements in the promoter region mainly suggested that these *Osa-miR156j* played a critical role in the regulation of transcription factors, metabolisms, stress responses, signaling transductions, cellular structural components, and other cellular processes.

## Conclusion

Under arsenic abiotic stress, the expression analysis of *Osa-miR156j* has successfully indicated *Osa-miR156j* importance at various developmental stages and tissues in rice. Additionally, the target functions and presence of oxidative stress-responsive *cis*-acting regulatory elements/motifs also confirmed the *miR156j* involvement in the regulation of arsenic abiotic stress, which is highly influenced by exposure duration and plant tissues.

## Data Availability Statement

All datasets generated for this study are included in the article/Supplementary Material.

## Author Contributions

AV conceived grant-in-aid, designed, and supervised the complete study and supervised the experimental procedure and proofread the entire manuscript. MG and AP performed all the wet and dry lab experiments and statistical analysis besides draft preparation. All authors contributed to the manuscript and approved the submitted version.

## Conflict of Interest

The authors declare that the research was conducted in the absence of any commercial or financial relationships that could be construed as a potential conflict of interest.

## Footnotes

1 http://plantgrn.noble.org/psRNATarget/

2 http://bioinfo.cau.edu.cn/agriGO/

3 http://bioinformatics.psb.ugent.be/webtools/plantcare/

4 http://ricefrend.dna.affrc.go.jp/multi-guide-gene.html

5 https://biit.cs.ut.ee/clustvis/

## Supplementary Material

TABLE S1 Primers and probes used in the study.

TABLE S2 *Osa-miR156j* potential target genes, locus IDs, and their target functions during arsenic abiotic stress.

TABLE S3 *Osa-miR156j* target gene; Os01g0922600, Os06g0663500, Os07g0505200Os09g0507100, Os02g0174100, Os09g0513100, and their potential co-expressed target genes, functions in the co-expression network.

TABLE S4 Indicates the data obtained from the rice Massively Parallel Signature Sequencing database with both 17 and 20 bp sequences for different development stages and various tissues in rice.
