# Supplementary material for: Effect of Arsenic Stress on Expression Pattern of a Rice Specific miR156j at Various Developmental Stages and Their Allied Co-expression Target Networks
Source: Front Plant Sci. 2020 Jun 16;11:752. doi: 10.3389/fpls.2020.00752 (PMC7308582; doi:10.3389/fpls.2020.00752)
Supplement: TABLE S1 — Primers and probes used in the study. [file Table_1.DOCX]

|  | **Forward primer** | **Reverse Primer** |
| --- | --- | --- |
| Osa-miR156j | GCAGTGACAGAAGAGAGTGT | AGTTTTTTTTTTTTTTTTGTGCT |
| Actin-1 | ACTCTCGCCTCCTCGCGAC | AGTAGCTTACATGACAAGGGCT |
| Osa-miR156j Probe | TGCTCACTCTCTTCTGTCA | |
