# Supplementary material for: Effect of Arsenic Stress on Expression Pattern of a Rice Specific miR156j at Various Developmental Stages and Their Allied Co-expression Target Networks
Source: Front Plant Sci. 2020 Jun 16;11:752. doi: 10.3389/fpls.2020.00752 (PMC7308582; doi:10.3389/fpls.2020.00752)
Supplement: TABLE S2 — Osa-miR156j potential target genes, locus IDs, and their target functions during arsenic abiotic stress. [file Table_2.docx]

| **miRNA accession** | **Target genes** | **Locus ID** | **Gene annotation** | **Target function** |
| --- | --- | --- | --- | --- |
| Os-miR156j | Os09g31438 | - | Squamosa promoter binding like protein 9 | DNA binding transcription factor |
|  | Os08g39890 | Os08g0509600 |  |  |
|  | Os02g07780 | Os02g0174100 | Squamosa promoter- binding- like protein 10 | DNA binding transcription factor |
|  | Os02g04680 | Os02g0139400 |  |  |
|  | Os06g45310 | Os06g0663500 | Squamosa promoter- binding- like protein 11 | DNA binding transcription factor |
|  | Os07g32170 | Os07g0505200 | SBP domain containing protein | DNA binding transcription factor |
|  | Os11g30370 | - | Teosinte glume architecture 1 | DNA binding transcription factor |
|  | Os09g32944 | Os09g0507100 |  |  |
|  | Os01g69830 | Os01g0922600 |  |  |
|  | Os09g33820 | Os09g0513100 | Phospholipase A1 expressed protein | Phospholipase A1 activity |
|  | Os06g43562 | - | Acetyltransferases expressed protein | Acetyltransferase activity |
|  | Os09g03670 | - | Retrotransposon protein | DNA binding proteins |
|  | Os08t050960 | - | SPL transcription activator (Branching in panicle and vegetative shoot regulator) | DNA binding transcription factor |
|  | Os08t053160 | - | Transcription factor (Positive regulator of cell proliferation) | DNA binding transcription factor |
